# Genetics of enzymatic dysfunctions in metabolic disorders and cancer

**DOI:** 10.3389/fonc.2023.1230934

**Published:** 2023-08-02

**Authors:** Mélanie Mahé, Tiffany J. Rios-Fuller, Andrea Karolin, Robert J. Schneider

**Affiliations:** Department of Microbiology, Grossman NYU School of Medicine, New York, NY, United States

**Keywords:** inherited metabolic disorders, enzymatic dysregulation, cancer, urea cycle, glycogen storage, lysosome storage, fatty acid oxidation, mitochondrial respiration

## Abstract

Inherited metabolic disorders arise from mutations in genes involved in the biogenesis, assembly, or activity of metabolic enzymes, leading to enzymatic deficiency and severe metabolic impairments. Metabolic enzymes are essential for the normal functioning of cells and are involved in the production of amino acids, fatty acids and nucleotides, which are essential for cell growth, division and survival. When the activity of metabolic enzymes is disrupted due to mutations or changes in expression levels, it can result in various metabolic disorders that have also been linked to cancer development. However, there remains much to learn regarding the relationship between the dysregulation of metabolic enzymes and metabolic adaptations in cancer cells. In this review, we explore how dysregulated metabolism due to the alteration or change of metabolic enzymes in cancer cells plays a crucial role in tumor development, progression, metastasis and drug resistance. In addition, these changes in metabolism provide cancer cells with a number of advantages, including increased proliferation, resistance to apoptosis and the ability to evade the immune system. The tumor microenvironment, genetic context, and different signaling pathways further influence this interplay between cancer and metabolism. This review aims to explore how the dysregulation of metabolic enzymes in specific pathways, including the urea cycle, glycogen storage, lysosome storage, fatty acid oxidation, and mitochondrial respiration, contributes to the development of metabolic disorders and cancer. Additionally, the review seeks to shed light on why these enzymes represent crucial potential therapeutic targets and biomarkers in various cancer types.

## 1 Introduction

**Figure 1:**
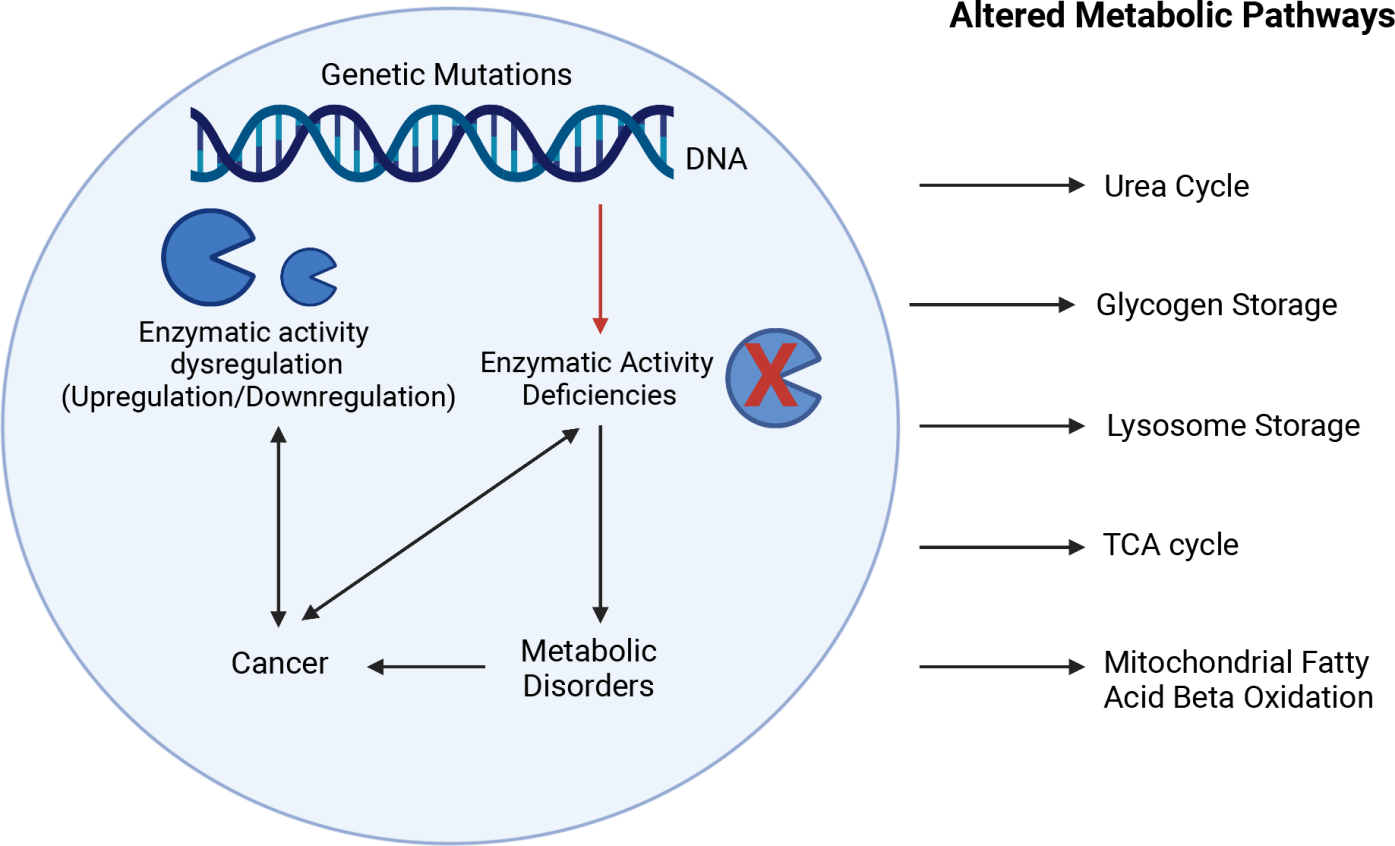
Interplay between enzymatic deficiencies, metabolic disorders, and cancer. Enzymatic activity deficiencies due to genetic mutations or changes in levels of expression (upregulation and downregulation) can lead to several metabolic disorders and have been associated with cancer development affecting metabolic pathways such as urea cycle, glycogen storage, lysosome storage, fatty acid oxidation, and mitochondria respiration. The Figure was partly generated using Biorender under the agreement number: WM25LGLSAK (www.Biorender.com).

Inherited metabolic disorders can be caused by mutations of genes involved in the biogenesis, assembly or activity of metabolic enzymes, which can lead to enzymatic deficiency and severe life-threatening metabolic impairments (1). Metabolism is the process by which macromolecules (lipids, carbohydrates, nucleic acids, and proteins) are broken down to produce energy (catabolism) or used for energy storage (anabolism). In normal cells, macromolecules go through a series of biochemical reactions catabolized by metabolic enzymes in the presence of oxygen to produce ATP through mitochondrial respiration. By-products resulting from this metabolic activity are then recycled or eliminated. Dysregulation of the activity of these enzymes due to mutation or changes in levels of expression (upregulation and downregulation) can lead to several metabolic disorders and also have been associated with cancer development (**Figure 1**) (2).

The “Warburg effect” or aerobic glycolysis, one of the altered metabolisms in cancer, was first described in 1927 by Otto Warburg who observed that cancer cells have altered glucose metabolism due to increased glucose uptake in the cytoplasm where glucose is converted into lactate, even in the presence of oxygen (3). This discovery showed for the first time how cancer cells can benefit from metabolic adaptation to ensure their survival and proliferation. It has been proposed that cancer cells, which require high energy consumption, use aerobic glycolysis to facilitate the uptake and incorporation of nutrients into their biomass (4). However, the reasons why some cancer cells switch from oxidative phosphorylation to aerobic glycolysis remain unclear (5), and recent research has shown that oxidative phosphorylation can also drive cancer growth (6). Since the “Warburg effect” discovery, considerable research has been conducted on the importance of metabolism for cancer development, making metabolism reprogramming one of the hallmarks of cancer and cancer itself a metabolic disease (7, 8). Cancer cell rewiring of metabolism plays a key role in tumorigenesis, tumor progression, and drug resistance, which can be influenced by the tumor microenvironment (TME) and the genetic context in which tumors arise and progress. Enzymatic deficiency can notably lead to an accumulation of metabolites, known as oncometabolites, which can act as signaling molecules for regulating gene expression and promoting tumor growth (9–11).

The upregulation or downregulation of metabolic enzymes can promote and sustain the activation of metabolic pathways that play a key role in cancer cell proliferation and survival, notably by preventing nutrient depletion (2). However, whether the expression of metabolic enzymes is a cause or a consequence of metabolic adaptations often remains unclear. As the interplay between cancer and metabolism reprogramming is becoming established, more research is needed to fully understand how cancer cells take advantage of metabolic enzyme dysregulation. In this context, here we review enzymatic dysregulation of the metabolic pathways for the urea cycle, glycogen storage, lysosome storage, fatty acid oxidation and mitochondrial respiration, with regard to their role in the development of metabolic disorders and cancer, and why these enzymes represent important potential therapeutic targets and biomarkers in most cancer types.

## 2 Urea cycle disorders

**Figure 2:**
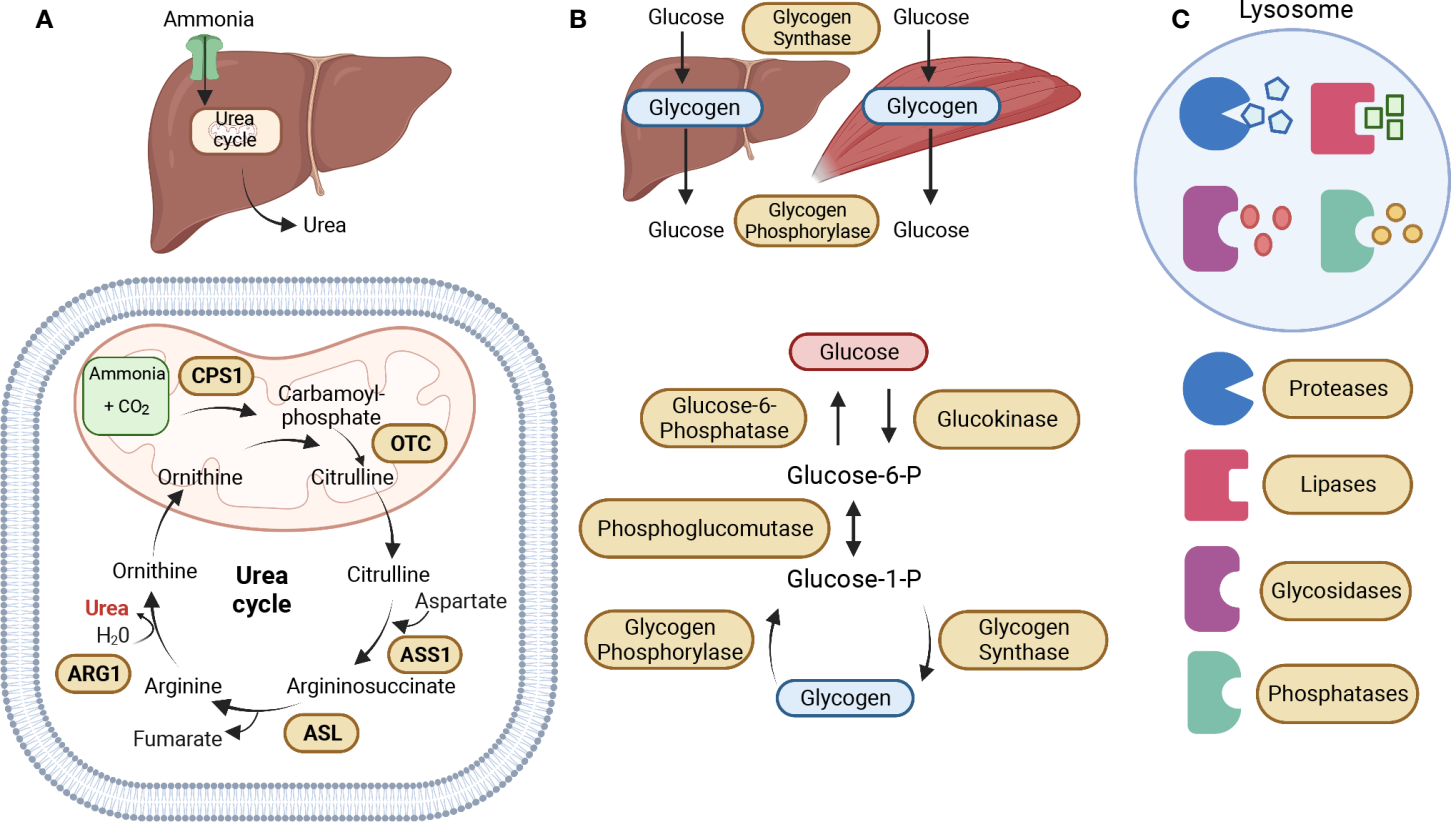
Metabolic Enzymatic Pathways. **(A)** The urea cycle is the primary pathway for the elimination of nitrogenous waste, mainly in the liver, such as ammonia and glutamine, into urea. The five catalytic enzymes in the urea cycle are Carbamoyl phosphate synthetase I (CPS1), Ornithine transcarbamylase (OTC), which are both located in the mitochondrial matrix, Argininosuccinate synthetase (ASS1), Argininosuccinate lyase (ASL), and Arginase (ARG1), located in the cytoplasm. The urea cycle starts in the mitochondrial matrix with the conversion of ammonia into carbamoyl-phosphate, which is then converted into citrulline by OTC. Citrulline is exported to the cytoplasm where it is converted into argininosuccinate by ASS1. The ASL enzyme then converts argininosuccinate into arginine, which is then converted into ornithine by ARG1, leading to the production of urea. Ornithine enters the mitochondria to participate in the conversion of carbamoyl phosphate into citrulline. **(B)** Glycogen serves as the main storage form of glucose in humans, mostly in the liver and muscles. The primary enzymes involved in glycogen synthesis (glycogenesis) and breakdown (glycogenolysis) are glycogen synthase and glycogen phosphorylase. During glycogenesis, glucose is converted into glucose-6-phosphate (Glucose-6-P) by glucokinase. Glucose-6-P is converted into glucose-1-phosphate (Glucose-1-P) by phosphoglucomutase. Then, Glucose-1-P is converted to glycogen by the enzyme glycogen synthase. During glycogenolysis, glycogen is converted to Glucose-1-P by glycogen phosphorylase, which is then converted back into Glucose-6-P by phosphoglucomutase. And finally, Glucose-6-P is converted to glucose by Glucose-6-phosphatase. **(C)** The lysosome is an essential catabolic organelle that provides an acidic environment, where macromolecules are metabolized by hydrolytic enzymes, such as proteases, lipases, glycosidases, and phosphatases. The Figure was partly generated using Biorender under the agreement number: TR25LH0BWF (www.Biorender.com).

Urea cycle defects or disorders (UCDs) arise from an inherited deficiency in one of the five catalytic enzymes that play a crucial role in the urea cycle pathway. This leads to an accumulation of ammonia (hyperammonemia), which in turn results in neurocognitive deficits and/or chronic liver dysfunction. The urea cycle is the primary pathway for the elimination of nitrogenous waste, mainly in the liver, such as ammonia and glutamine, into urea (12). The five catalytic enzymes in the urea cycle are Carbamoyl phosphate synthetase I (CPS1), Ornithine transcarbamylase (OTC), Argininosuccinate synthetase (ASS1), Argininosuccinate lyase (ASL), and Arginase (ARG1) (**Figure 2A**) (13). The manifestation of deficiency in any of these enzymes has been linked with the progression of cancer due to the generation of nucleotide imbalances that instigate the occurrence of mutation patterns. This highlights the importance of these enzymes in maintaining normal cellular function and preventing the development of cancer.

The first step in the urea cycle is catalyzed by CPS1, converting ammonia into carbamoyl phosphate (CP). CPS1 deficiency is characterized by complete or partial absence of the CPS enzyme, leading to patients experiencing vomiting, seizures, progressive lethargy, coma, and even death (14). CPS1 overexpression has been linked to poor prognosis in various types of cancer, including colorectal (15), cholangiocarcinoma (16), glioblastoma (17), lung adenocarcinoma (18), and non-small cell lung cancer (NSCLC) (19, 20). Upregulated CPS1 expression in tumor cells produces significant amounts of CP, which is then translocated to the cytoplasm and incorporated into the reaction catalyzed by a trifunctional enzyme, the CAD protein (21, 22). CAD is composed of carbamoyl-phosphate synthetase 2, aspartate transcarbamylase, and dihydroorotase, necessary to maintain cellular fundamental function (i.e., DNA and RNA biosynthesis) by initiating pyrimidine synthesis (23). However, in other types of cancer, such as small intestine adenocarcinoma (24) and hepatocellular carcinoma (HCC) (25), the levels of CPS1 are downregulated, which associates with decreased survival and an increase of CAD expression, resulting in the reuse of ammonia for the synthesis of glutamine as a means to initiate *de novo* pyrimidine synthesis (21).

The second step in the urea cycle is catalyzed by OTC, converting ornithine and carbamoyl phosphate into citrulline, which detoxifies the ammonia produced from amino acid catabolism. OTC deficiency is a rare X-linked genetic disorder identified by complete or partial lack of the enzyme OTC, leading to impairment of the central nervous system, which has the potential to result in permanent brain damage and is fatal in newborn infants (26). The downregulated OTC expression level results in accumulated ammonia and has been associated with larger tumor size, advanced grade, and poor prognosis for patients with hepatocellular carcinoma (HCC) (27). The downregulation of mitochondrial NAD-dependent protein deacetylase sirtuin-3 (SIRT3) in HCC cells may contribute to the protection of these cells from apoptosis. SIRT3 is a regulator of OTC deacetylation, and the acetylation of lysine 88 inhibits the enzyme activity of OTC, highlighting the important role of a deacetylase in regulating the function of OTC (26, 28, 29).

The third step in the urea cycle is catalyzed by ASS1, in which citrulline is condensed with aspartate to form argininosuccinic acid and functions as an enzyme for arginine metabolism (30). Citrullinemia type I (CTLN1) is caused by a deficiency or absence of the enzyme ASS1, resulting in increased intracranial pressure (ICP), increased neuromuscular tone, seizures, loss of consciousness, and death (31). The incidence of ASS1 deficiency changes significantly with the tumor type and tissue of origin (32). Increased levels of ASS1 have been observed in human non-small cell lung cancer (NSCLC) and colon carcinomas, which may be supporting arginine synthesis and facilitating cellular survival under low-nutrient stress conditions (33). In contrast, decreased ASS1 levels have been shown in breast cancer, primary hepatocellular carcinoma (HCC), melanoma, sarcomas, renal cell carcinoma, and prostate cancer (32). ASS1 loss in tumors hinders arginine biosynthesis, leading to dependence on extracellular arginine for survival. Thus, arginine depletion therapy is a promising strategy for ASS1-negative tumors, which constitute nearly 70% of tumors (30). Rabinovich S et al. were also able to demonstrate that ASS1 deficiency in cancer increases cytosolic aspartate levels leading to increased activation of the enzymatic complex CAD (carbamoyl-phosphate synthase 2, aspartate transcarbamylase, dihydroorotase complex) by upregulating its substrate availability and by increasing its phosphorylation by S6K1 through the mTOR pathway. They were able to show that decreased activity of ASS1 in cancers supports proliferation by activating CAD and facilitating pyrimidines synthesis (34). Furthermore, ASS1 plays a crucial role as a biomarker for the response to glutamine deprivation. Impairment of ASS1 activity elevates sensitivity towards arginine and glutamine deprivation, whereas upregulation of ASS1 activity augments resistance towards arginine and glutamine deprivation (35).

The fourth reaction in the urea cycle is catalyzed by ASL, leading to the breakdown of argininosuccinic acid to arginine and fumarate. Argininosuccinic aciduria is an inherited disorder described by deficiency or lack of the enzyme ASL, leading to an accumulation of citrulline and argininosuccinic acid, causing vomiting, drowsiness, seizures, and/or coma (36). ASL is highly expressed in melanoma, HCC, and breast tumor tissues (37, 38). ASL and nitric oxide synthase (NOS) form the citrulline-argininosuccinate-arginine cycle, facilitating nitric oxide (NO) production. Overproduction of NO has been associated with the progression of cancer (38, 39).

The fifth reaction in the urea cycle is catalyzed by ARG1, involved in the hydrolysis of arginine to ornithine and urea, which regulate the proliferation, differentiation, and function of different cell types. Arginase-1 deficiency is identified by either a complete or partial absence of the arginase enzyme in the liver and red blood cells, with symptoms that can include vomiting, poor growth, seizures, and stiff muscles with increased reflexes (spasticity) (40, 41). Increased expression of arginases (either Arg1 or Arg2) is considered a poor prognostic factor in several types of cancer, including lung cancer (42, 43), head and neck cancer (44), neuroblastoma (45), acute myeloid leukemia (46), pancreatic ductal carcinoma (47), ovarian carcinoma (48), and colorectal cancer (49). Arginine metabolism plays a crucial role in T-cell activity and survival. Increased enzymatic activity of arginase depletes arginine levels in the tumor microenvironment, leading to immunosuppression and impaired T-cell function, which is critical for effective immune surveillance and anti-tumor response (50).

The urea cycle and its five catalytic enzymes play a crucial role in maintaining normal cellular function, and their deficiencies have been associated with cancer progression. **Table 1** provides a comprehensive overview of enzyme mutations in the urea cycle, their respective enzymatic roles, associated diseases, and the regulation status (up or down) of both the enzyme and genes in different types of cancer. Further research is needed to explore the interplay between the urea cycle and cancer progression. Understanding the molecular mechanisms underlying these defects may provide potential targets for therapeutic interventions to prevent cancer development and improve patient outcomes.

**Table 1:** Urea Cycle Disorders (UCDs).

Enzymes	Role	Disease Name	Upregulated Cancers	Downregulated Cancers
Carbamoyl phosphate synthetase I (CPS1)	Synthesizes carbamoyl phosphate (CP) from ammonia, bicarbonate, and 2 molecules of ATP.	Carbamoyl phosphate synthetase I deficiency (CPS1 deficiency)	Colorectal (15), cholangiocarcinoma (16), glioblastoma (17), lung adenocarcinoma (18), and non-small cell lung cancer (NSCLC) (19, 20).	Small intestine adenocarcinoma (24) and hepatocellular carcinoma (HCC) (25).
Ornithine transcarbamylase (OTC)	Catalyzes the reaction between CP and ornithine to form citrulline and phosphate.	Ornithine transcarbamylase deficiency (OTC deficiency)		HCC (27).
Argininosuccinate synthetase (ASS1)	Catalyzes the synthesis of argininosuccinic acid from citrulline and aspartate	Arginosuccinate synthetase deficiency (ASS deficiency), also known as Citrullinemia type I (CTLN1)	NSCLC and colon carcinomas (33).	Breast, HCC, melanoma, sarcomas, renal cell carcinoma, and prostate (32).
Argininosuccinate lyase (ASL)	Catalyzes the reversible hydrolytic cleavage of argininosuccinic acid into arginine and fumarate	Argininosuccinate lyase deficiency (ASL deficiency), also known as Argininosuccinic aciduria	Melanoma, HCC, and breast (37, 38).	
Arginase (ARG1)	Catalyzes the breakdown of arginine into urea and ornithine	Arginase deficiency (ARG1 deficiency), also known as Argininemia or Hyperargininemia	Lung (42, 43), head and neck cancer (44), neuroblastoma (45), acute myeloid leukemia (46), pancreatic ductal carcinoma (47), ovarian carcinoma (48), and colorectal (49).	

## 3 Glycogen storage disorders

Glycogen Storage Disorders (GSDs) are a set of hereditary metabolic disorders that affect glycogen metabolism, which is responsible for regulating glycogen synthesis or degradation (51). Glycogen serves as the main storage form of glucose in humans, mostly in the liver and muscles (52). The primary enzymes involved in glycogen synthesis (glycogenesis) and breakdown (glycogenolysis) are glycogen synthase and glycogen phosphorylase (**Figure 2B**) (51). GSDs are classified based on the specific enzyme deficiency and the primary affected tissues with an increasing number of GSD types being identified. We will be focusing on Type 0 due to its two distinct forms of glycogen synthase, and Types I, II, III, and IV, which are the four most common types of GSD (53). **Table 2** describes the mutated names of enzymes involved in glycogen storage, alongside their enzymatic roles and the corresponding diseases they are associated with, and information about whether the enzymes or genes are upregulated or downregulated in various types of cancer.

**Table 2:** Glycogen Storage Disorders (GSDs).

Enzymes	Role	Disease Name	Upregulated Cancers	Downregulated Cancers
Glycogen synthase	Catalyzes the rate-limiting step in glycogenesis by transferring glucose monomers to growing glycogen chains	GSD Type 0, also known as Glycogen synthase deficiency (Muscle glycogen synthase deficiency (encoded by *GYS1*) and liver glycogen synthase deficiency (encoded by *GYS2*)).	*GYS1* in glioblastoma, breast, and colon (54).	*GYS2* in HCC (55).
Glucose-6-Phosphatase (G6Pase)	Provides glucose during starvation by catalyzing the hydrolysis of glucose-6-phosphate (G6P).	GSD Type I or Von Gierke disease, also known as Glucose-6-phosphate deficiency. GSD Type 1a (GSD1a) glucose-6-phosphatase (G6Pase) deficiency (encoded by *G6PC*). Type 1b and 1c (GSD1b or 1c) glucose-6-phosphate translocase (G6PT) deficiency (encoded by *SLC37A4*).	*G6PC* in ovarian (56), glioblastoma (57), and cervical (58).	*G6PC* in HCC (59) and clear renal cell carcinoma (60).
alpha-1-4-glucosidase (acid maltase)	Breaks down glycogen into glucose in the lysosome.	GSD Type II or Pompe disease, also known as alpha-1,4-glucosidase deficiency.		Pancreatic cells (61).
Glycogen debranching enzyme	Breaks down glycogen and mobilizes glucose reserves from glycogen deposits in the muscles and liver.	GSD Type III, Cori disease or Forbes disease, also known as Glycogen debrancher deficiency.		Bladder (62).
Glycogen branching enzyme	Adds branches to the growing glycogen molecule during glycogenesis	GSD Type IV or Andersen disease, also known as Glycogen branching enzyme deficiency.	Lung adenocarcinoma (63–65).	

GSD type 0 is caused by mutations in the *GYS1* gene, leading to muscle glycogen synthase deficiency, and mutations in the *GYS2* gene, leading to liver glycogen synthase deficiency. The two isoforms of glycogen synthase share a common role of forming glycogen by linking glucose molecules (66). A study by Favaro et al. showed that *GYS1* is rapidly induced in glioblastoma, breast, and colon cancer cell lines under hypoxic conditions, followed by a decrease of glycogen phosphorylase (PYGL), an enzyme that degrades glycogen. This results in glycogen accumulation, decreased nucleotide synthesis, and increased reactive oxygen species (ROS) levels that contribute to p53-dependent growth arrest and impaired tumorigenesis *in vivo* (54). Meanwhile, the knockdown of *GYS2* in HCC promotes cell proliferation *in vitro* and tumor growth *in vivo* by regulating p53 expression. Interestingly, p53 is capable of transcriptionally regulating *GYS2*, *PYGL*, and other genes involved in glycogen synthesis (55). In addition, p53 has been identified as a key regulator of glucose metabolism through its ability to suppress glucose uptake and glycolysis in tumor cells (67).

GSD type I, also known as Von Gierke disease, has three subtypes: GSD 1a is caused by *G6PC* gene mutations involving glucose-6-phosphatase (G6Pase) deficiency. G6Pase is a membrane-bound protein associated with the endoplasmic reticulum (ER) involved in providing glucose during starvation by catalyzing the hydrolysis of glucose-6-phosphate (G6P) (68). While GSD 1b and 1c are caused by *SLC37A4* gene mutations resulting in glucose-6-phosphate translocase (G6PT) deficiency. G6PT is a transmembrane protein involved in translocating G6P from the cytosol into the lumen of the ER for glucose hydrolysis (69). Abnormal expression of *G6PC* is observed in different cancers, with low expression in HCC (59) and clear renal cell carcinoma (60), likely resulting in the accumulation of G6P. This accumulation of G6P may lead to increased glucose metabolism by producing ribose-5-phosphate through the hexose monophosphate (HMP) shunt pathway (an alternative pathway to glycolysis) in tumor cells, resulting in cell division, cell survival, and tumor growth (59). In contrast, overexpression of *G6PC* affects glucose metabolism in ovarian (56), glioblastoma (57), and cervical cancer (58), contributing to tumor proliferation, metastasis, and poor prognosis in patients. The overexpression of *G6PC* increases the amount of blood glucose, leading to an increase in the rate of glycolysis. This may be occurring by inducing alteration in other pathways, such as cell cycle regulation via the Forkhead box protein O1 (FOXO1) pathway in ovarian cancer (56), intracellular glycogen degradation by hypoxia-inducible factor 1-alpha (HIF1α) and signal transducer and activator of transcription 3 (STAT3) in glioblastoma (57), and by regulating the activation of PI3K/AKT/mTOR pathway in cervical cancer (58). In turn, *G6PT* regulates glucose homeostasis in glioblastoma leading to inhibition of cancer cell proliferation, extracellular matrix (ECM) degradation, or inducing cell death. *G6PT* may be functioning as a “bioswitch” allowing cells to switch between migration or cell death in response to external stimuli, such as hypoxia or intracellular metabolic changes (i.e., Ca^2+^ flux and cytosolic ATP) controlled by the PTEN/Akt/PI3K/mTOR pathway (70). Furthermore, overexpression of *G6PT* in glioblastoma cells induced cell migration by regulating calcium-mediated signaling (71) and *G6PT* expression regulates bone marrow-derived stromal cells (BMSC) survival, ECM degradation, and mobilization by inhibiting the activation of pro-matrix metalloproteinase-2 (proMMP-2) mediated by membrane type 1 matrix metalloproteinase (MT1-MMP) (72).

GSD type II (known as Pompe disease), also classified as lysosomal storage disease, is caused by mutations in the *GAA* gene resulting in a deficiency of alpha-1-4-glucosidase (acid maltase) causing marked accumulation of glycogen in lysosomes (73). Hamura et al. showed that knockdown of *GAA* decreased cell proliferation and increased apoptotic signals in pancreatic cells, accompanied by accumulation of dysfunctional mitochondria, caused by the suppression of the transcription factor EB (TFEB), which plays a critical role in lysosomal biogenesis (61).

GSD type III, also known as Cori or Forbes disease, is caused by mutations in the *AGL* gene, which results in glycogen debranching enzyme deficiency, an enzyme that helps facilitate the breakdown of glycogen and mobilize glucose reserves from glycogen deposits in the muscles and liver. A recent study by Guin et al. showed that *AGL* serves as a prognostic marker for bladder cancer survival, and decreased *AGL* enhances tumor growth by increasing glycine synthesis through increased expression of serine hydroxymethyltransferase 2 (SHMT2), an enzyme that allows cells to process glycogen into glycine (62).

GSD type IV, also known as Andersen disease, results from mutations in the *GBE1* gene, causing a deficiency in the glycogen branching enzyme, which adds branches to the growing glycogen molecule during the synthesis of glycogen, allowing for easy and quick glycogen utilization when it is broken down (74). Studies have revealed that *GBE1* expression is upregulated in hypoxia-conditioned primary lung adenocarcinoma cells mediated by HIF1α, while decreased *GBE1* expression inhibits lung cancer cell growth by directly affecting glycogen production and glucose metabolic signaling pathways. These findings suggest that *GBE1* expression protects cells from hypoxia and allows them to survive, thereby further promoting proliferation and metastasis (63–65).

Glycogen accumulation has been shown to play a crucial role in promoting cell survival under hypoxic conditions in both normal and cancer cells, as demonstrated by various studies including cancer cell lines such as breast, kidney, uterus, bladder, ovary, skin, and brain cancer cell lines (54, 64, 75–79). Furthermore, a recent study found that glycogen accumulation is essential for tumor initiation in human and mouse liver tumors, which commonly exhibit hypoxic stress in the early stages (80). The elimination of glycogen accumulation has been shown to abrogate liver cancer incidence, while increasing glycogen storage accelerates tumorigenesis. These findings suggest that glycogen metabolism plays a crucial role in tumor initiation and growth and could be a potential target for cancer treatment.

## 4 Lysosomal storage disorders

Lysosome Storage Disorders (LSDs) are caused by heritable mutations in genes encoding lysosomal enzymes (known as hydrolytic enzymes), resulting in the buildup of various unmetabolized macromolecules (i.e., proteins, lipids, carbohydrates, and nucleic acids) impairing lysosomal homeostasis and activity (81). The lysosome is an essential catabolic organelle found in eukaryotic cells and provides an acidic environment, where macromolecules are metabolized by hydrolytic enzymes, such as glycosidases, lipases, proteases, sulfatases, nucleases, and phosphatases (**Figure 2C**) (82, 83). Hydrolytic enzymes facilitate the breakdown of chemical bonds within different types of compounds including proteins, nucleic acids, starch, fats, phosphate esters, and other macromolecules (84). LSDs represent over 70 disorders, characterized by lysosomal dysfunction, in which 50 of these disorders are caused by enzyme deficiencies. **Table 3** offers insights into enzyme mutations and their respective enzymatic functions in lysosome storage, along with associated diseases. Additionally, it highlights whether the enzymes or their corresponding genes are upregulated or downregulated in different types of cancer. Depending on the accumulated material in the lysosomes, these enzyme deficiencies can be classified into three categories: sphingolipidoses, glycoproteinosis, and mucopolysaccharidoses (118).

**Table 3:** Lysosome Storage Disorders (LSDs).

Sphingolipidoses				
Enzymes	Role	Disease Name	Upregulated Cancers	Downregulated Cancers
β-Glucocerebrosidase (β-glucosidase) encoded by *GBA*	Breaks down glucocerebroside into glucose and ceramide.	Gaucher Disease (GD), also known as Glucocerebrosidase deficiency		*GBA* in liver (85).
α-Galactosidase A	Breaks down globotriaosylceramide (known as Gb3 or CD77)	Fabry disease, also known as Alpha-galactosidase A deficiency	Gb3 in breast (86, 87), ovarian (88), and colon cancer (89).	
Acid ceramidase	Metabolizes ceramides into sphingosine and a fatty acid	Farber disease, also known as Farber lipogranulomatosis or Acid ceramidase deficiency	Prostate cancer (90, 91), head and neck squamous cell carcinoma (92), liver (93), and breast (94).	
Acid sphingomyelinase (ASM)	Metabolizes the hydrolysis of sphingomyelin into phosphorylcholine and ceramide.	Niemann-Pick Disease Types A and B (NPD-A and B), also known as Sphingomyelinase deficiency		Breast, lung, thyroid, and bladder (95).
Glycoproteinoses				
Enzymes	Role	Disease Name	Upregulated Cancers	Downregulated Cancers
Lysosomal α-mannosidase, encoded by *MAN2B1*	Breaks down oligosaccharides containing a mannose.	α-mannosidosis, also known as Alpha-mannosidase deficiency or Mannosidosis	*MAN2B1* in bladder urothelial carcinoma, breast invasive carcinoma, colon adenocarcinoma, glioblastoma multiforme, low-grade gliomas, and laryngeal cancer (96, 97).	
α-L-fucosidase, encoded by *FUCA1*	Cleaves fucose-rich oligosaccharides, glycoproteins, and glycolipids.	Fucosidosis, also known as Alpha-fucosidase deficiency	*FUCA1* in glioblastoma multiforme (98), papillary thyroid cancer (PTCs) samples (99), and breast cancer (100).	*FUCA1* in colorectal cancer (101), HCC (102), and anaplastic thyroid cancer (ATCs) samples (99).
Lysosomal neuraminidase-1 (NEU1; also known as sialidase)	Removes terminal sialic acid residues from sialo-rich oligosaccharides, glycoproteins and glycolipids.	Sialidosis, also known as Mucolipidosis Type I	HCC (103, 104), ovarian (105), and colon (106).	
Mucopolysaccharidoses				
Enzymes	Role	Disease Name	Upregulated Cancers	Downregulated Cancers
Alpha-L-iduronidase (IDUA)	Breaks down glycosaminoglycans, such as dermatan sulfate and heparan sulfate.	MPS I, also known as IDUA deficiency, Hurler syndrome, Scheie syndrome or Hurler-Scheie syndrome		Breast (107) and ovarian (108).
Iduronate-2-sulfatase (IDS)	Breaks down glycosaminoglycans, such as dermatan sulfate and heparan sulfate.	MPS II, also known as Hunter Syndrome or Iduronate 2-sulfatase deficiency		Breast (109).
Arylsulfatase B (ARSB; also known as N−acetylgalactosamine−4−sulfatase)	Breaks down glycosaminoglycans, such as dermatan sulfate and chondroitin sulfate.	MPS VI, also known as Maroteaux-Lamy syndrome or Arylsulfatase B deficiency		Melanoma (110), colorectal (111), prostate, and breast (112, 113).
β−glucuronidase, encoded by *GUSB*	Breaks down glycosaminoglycans, such as dermatan sulfate and keratan sulfate.	MPS VII, also known as Sly syndrome or Beta-glucuronidase deficiency	Colorectal (114), gastric (115), and pancreas (116). *GUSB* in HCC (117).	

### 4.1 Sphingolipidoses

Sphingolipidoses are a group of heterogeneous inherited metabolic disorders characterized by an accumulation of glycolipids or phospholipids, which have ceramide as a common structure (119). Sphingolipidoses can lead to several diseases, the most common are Gaucher’s disease (GD), Fabry disease, Farber disease, and Niemann-Pick disease.

The most common LSDs is Gaucher Disease (GD), an autosomal recessive disorder caused by mutations in the *GBA* gene, resulting in β-Glucocerebrosidase (β-glucosidase) deficiency. β-glucosidase is an enzyme that helps break down glucocerebroside into glucose and ceramide. Deficiency of β-glucosidase leads to an accumulation of glucocerebroside (also called glucosylceramide) and glucosylsphingosine in macrophages through the body, called Gaucher cells, mainly affecting the liver, spleen, and bone marrow (120, 121). GD has been classified by type and severity of neurological involvement: Type 1 GD (GD1) is defined as the non-neuronopathic subclass, acute neuronopathic GD (GD2) is characterized by acute neurological decline, and chronic neuronopathic GD (GD3) is identified by a highly variable spectrum of associated neurological and non-neurological manifestations (122). According to the Gaucher Registry, GD1 is the most common accounting for 90% - 95% of all documented cases of GD in Europe and North America (123). Notably, several case reports have shown a link between patients with GD1 and different cancers, including bone (124), breast (125), colon (126, 127), hematologic (125, 128), kidney (125, 129), liver (125, 130, 131), melanoma (125), multiple myeloma (125, 132), and non‐Hodgkin lymphoma (125, 133). The development of cancer in GD patients could be explained by the accumulation of glucocerebroside in macrophages, leading to lipid-engorged macrophage activation, which affects immune system regulation in several different ways. The levels of pro-inflammatory, as well as, anti-inflammatory cytokines, chemokines, and growth factors, mostly those involved in inflammation and B-cell function are altered in the serum of GD patients compared to normal controls (134–138). The thymus shows the most prominent dysregulation, causing severe impairment of T-cell differentiation and maturation, abnormal B-cell recruitment, upregulation of CD1d and major histocompatibility complex (MHC) class II expression, which are mostly expressed on antigen-presenting cells (APCs), such as dendritic cells, thymic epithelial cells, and B cells. Suggesting impaired immune surveillance, which can support the development of malignancy (139–144). Furthermore, the downregulation of *GBA* expression in liver cancer tissues increased cellular glucosylceramide levels, promoting the metastasis ability by supporting the epithelial-mesenchymal transition (EMT) through activation of the Wnt/β-catenin signaling pathway (85).

Fabry disease is caused by mutations in the *GLA* gene leading to a deficiency of the α-Galactosidase An enzyme, causing the accumulation of globotriaosylceramide (known as Gb3 or CD77), a glycosphingolipid functioning as a receptor for pathogens and pathogenic products (145). This receptor particularly binds to the Shiga-like toxin 1 (SLT-1), a high-affinity harmless natural ligand that, upon binding to the receptor, the toxin is internalized and travels retrograde (against the flow) through the Golgi network and the ER, preventing the endo-lysosomal vesicular pathway, therefore, avoiding the degradation of the toxin (146, 147). Glycosphingolipids have been associated with oncogenesis (148). Several studies have shown that upregulation of Gb3 expression is necessary for cell invasiveness and correlates with metastasis in several cancer types, such as breast (86, 87), ovarian (88), and colon cancer (89). Suggesting that elevated Gb3 expression could act as an indicator of the transformation of tumor cells from their primary cancer state to the metastatic state, proposing that various invasive tumor types could share common mechanisms for metastasis (89). Moreover, a recent study showed that using the toxin internalization mechanism, they were able to deliver Shiga toxin-coated nanoparticles directly into the cytoplasm of Gb3-expressing head and neck cancer cells, demonstrating a novel way to deliver peptides or therapeutic nanomaterials inside cells (147).

Farber disease is a rare autosomal recessive disorder, also known as Farber lipogranulomatosis, caused by a mutation in the *ASAH1* gene, which leads to ceramide accumulation in several organs and tissues due to lysosomal acid ceramidase deficiency (149). Acid ceramidase is the enzyme that metabolizes ceramides into sphingosine and a fatty acid, products that are then recycled to create new ceramides. Ceramides are pro-apoptotic lipids and are part of the outer membrane surrounding cells, where they sense stress and other external factors and can mediate growth arrest, differentiation, and apoptotic cell death (150). In addition, acid ceramidase enzyme activity and sphingosine kinase can promote the formation of sphingosine-1-phosphate (S1P), a potent anti-apoptotic lipid mediating cell proliferation and survival (90). Acid ceramidase is overexpressed in prostate cancer (90, 91), head and neck squamous cell carcinoma (92), liver (93), and breast (94). The increase of acid ceramidase causes decreased ceramide accumulation and increased levels of sphingosine and S1P, indicating its involvement in metabolizing a significant portion of ceramides in tumor cells, resulting in tumor growth, survival, and resistance to therapy (90, 91, 93, 94). Suggesting that targeting the enzymes, acid ceramidase and sphingosine kinase, will block the tumor cell’s ability to metabolize ceramide, leading to an increase of pro-apoptotic ceramide levels, which will result in apoptosis (151, 152), growth inhibition (93, 152, 153), and increased sensitivity to radiation (154), and chemotherapeutics (155, 156).

Niemann-Pick Disease Types A and B (NPD-A and B) are rare autosomal recessive LSDs, categorized as sphingolipidoses due to sphingomyelin accumulation. These diseases arise from the deficiency of the acid sphingomyelinase (ASM) enzyme, caused by mutations in the sphingomyelin phosphodiesterase 1 (*SMPD1*) gene (157). ASM metabolizes the hydrolysis of sphingomyelin into phosphorylcholine and ceramide. A recent study demonstrated the incidence of cancer in patients diagnosed with ASM deficiency was abnormally elevated with four types of cancers being observed: breast, lung, thyroid, and bladder (95). Moreover, dysfunction of the ASM enzyme can alter sphingolipid metabolism leading to the downregulation of ceramide (a pro-apoptotic lipid) and the upregulation of S1P (a proliferative lipid) in cancer, possibly resulting in tumorigenicity and/or the potential to metastasize (158).

### 4.2 Glycoproteinoses

Glycoproteinoses are characterized as LSDs affecting glycoprotein degradation, causing an increased accumulation of undegraded oligosaccharides and/or glycoconjugates in lysosomes (159). Glycoproteinoses are rare and can lead to several diseases with high prevalence, such as α-mannosidosis, fucosidosis, and sialidoses (160).

α-mannosidosis is an autosomal recessive disorder caused by mutations in the *MAN2B1* gene, which encodes lysosomal α-mannosidase and results in α-mannosidase deficiency, leading to accumulation of mannose-rich oligosaccharides (161). Elevated expression of *MAN2B1* has been found in several cancers, including bladder urothelial carcinoma, breast invasive carcinoma, colon adenocarcinoma, glioblastoma multiforme, low-grade gliomas, and laryngeal cancer (96, 97). Specifically, the overexpression of *MAN2B1* in glioma tissues is associated with immune response and anti-inflammatory functions by correlating with the expression of tumor-associated macrophages and M2 macrophages, and correlates with malignant clinical features and poor outcome for glioma patients (96). In addition, expression of α-mannosidases has been shown in human papillomavirus (HPV)-associated cervical tumors (162) and nasopharyngeal carcinoma (163), resulting in tumor growth and metastasis. Furthermore, an inhibitor of α-mannosidases, known as swainsonine, was shown to block metastasis of melanoma and lymphoid tumor cells in mice and reduce the growth rate *in vitro* and *in vivo* of human melanoma cells. These data suggest that the expression of oligosaccharides associated with a malignant phenotype may be involved in tumor growth (164). However, other *in vivo* studies with HPV-associated cervical tumors, showed that swainsonine led to tumor growth, by inducing the accumulation of myeloid cells in the spleen of tumor-bearing mice, thereby inhibiting T-cell activation and aggravating the tumors system effects on the immune system, thus enabling tumor growth (162).

Fucosidosis is caused by mutations in the *FUCA1* gene, resulting in defective lysosomal α-L-fucosidase, which leads to the accumulation of fucose-rich oligosaccharides, glycoproteins, and glycolipids in tissues and urine (165). Several studies have shown that *FUCA1* is a p53 target gene, involved in tumorigenesis, and is capable of hydrolyzing various fucosylation sites on the epidermal growth factor receptor (EGFR), which ultimately determines the activation of EGFR (98, 99, 166, 167). According to various studies, it appears that the expression of *FUCA1* in human cancers is complex. Decreased *FUCA1* expression has been observed in colorectal cancer (101), hepatocellular carcinoma (102), and anaplastic thyroid cancer (ATCs) samples (99). While increased *FUCA1* expression has been observed in glioblastoma multiforme (98), papillary thyroid cancer (PTCs) samples (99), and breast cancer (100). Tsuchida et al., observed a potential relationship between *FUCA1* expression and p53 status, with a decreased expression of *FUCA1* and the presence of mutated p53 in ATCs, and an increased expression of *FUCA1* in PTCs, which predominantly harbor wild-type p53 (99). In addition, Ezawa et al. were able to demonstrate that tumor suppressor protein p53 is involved in protein glycosylation and targets *FUCA1* gene expression, resulting in its upregulation. This upregulation leads to the removal of fucose from the EGFR protein, ultimately suppressing cancer cell growth and inducing cell death. Furthermore, the study suggests that the upregulation of *FUCA1* expression contributes to the repression of the EGFR signaling pathway and has tumor‐suppressing activity in various human cancers (166). Moreover, Xu et al. showed that *FUCA1* is highly expressed in glioma tissues, leading to poor prognosis in glioma patients. The inhibition of *FUCA1* suppressed glioma growth *in vitro* and *in vivo*, promoting autophagy through the formation of large acidic vacuoles and by lowering levels of tumor-infiltrating macrophages (98).

Sialidosis, also known as Mucolipidosis Type I, is caused by autosomal recessive mutations in the *NEU1* gene, encoding the lysosomal enzyme neuraminidase-1 (NEU1; also known as sialidase), a glycosidase that removes terminal sialic acid residues from sialo-rich oligosaccharides, glycoproteins and glycolipids (168). Sialidase deficiency leads to the accumulation of sialyloligosaccharides and glycopeptides (169). *NEU1* is also involved in other cellular processes, such as cell proliferation/migration/differentiation, macrophage-associated immune and pro-inflammatory responses, and lysosomal exocytosis (103, 170–173). NEU1 is upregulated in HCC tumor tissues, which correlates with advanced stage, grade, and worse survival of HCC patients. Higher expression of NEU1 is associated with increased proliferation, migration, and lower levels of B cells, T-cells, and natural killer (NK) cells, regulating several tumor-related proteins and pathways, such as lysosome, spliceosome, and mTOR signaling pathways (103, 104). In pancreatic cancer cells, NEU1 forms a complex with MMP-9 and G protein-coupled receptors (GPCRs) to regulate EGFR activation and cellular signaling, playing a crucial role in the activation of receptor tyrosine kinases and downstream signaling pathways, making it a potential therapeutic target (174, 175). Oseltamivir phosphate (Tamiflu), anti-NEU1 antibodies, and broad-range MMP inhibitor galardin (GM6001) were found to inhibit NEU1 activity associated with EGF-stimulated cells (174). In addition, aspirin and celecoxib were also shown to inhibit NEU-1 activity in pancreatic cells, suggesting a novel multimodality mechanism of action for these drugs as anti-cancer agents (175). The inhibition of NEU1 activity in breast cancer cells, using oseltamivir phosphate or siRNA, also suppressed cell growth and induced apoptosis (176). Furthermore, NEU1 is overexpressed in ovarian cancer tissues compared with adjacent normal tissues. The siRNA of NEU1 in human ovarian cancer effectively inhibited proliferation, apoptosis, and invasion of cells by targeting lysosome and oxidative phosphorylation signaling (105). In contrast, NEU1 overexpression in colon cancer suppresses metastasis *in vivo*, and *in vitro* decreases cell migration, invasion, and adhesion, which involves downregulation of MMP-7, through integrin beta4-mediated signaling (106).

### 4.3 Mucopolysaccharidoses

Mucopolysaccharidosis (MPS) is an inherited disorder caused by a deficiency or malfunction in lysosomal enzymes responsible for breaking down glycosaminoglycans (GAGs), such as dermatan sulfate, heparan sulfate, keratan sulfate, and chondroitin sulfate. The ECM contains significant amounts of GAGs, which play a crucial role in promoting cell-to-cell and cell-to-ECM adhesion (177). The deficiency of the enzymes responsible for the proper degradation of GAGs can lead to systemic accumulation of GAGs in cells, blood, brain, spinal cord, and connective tissues. MPS is categorized as seven types of diseases, some of which are further categorized into subtypes. Six MPS types are autosomal-recessive inherited, and one type is inherited by the X-linked gene, known as MPS II or Hunter Syndrome (178). A retrospective study showed that the highest incidence rate at birth and prevalence rate was found for MPS I, II, and III in the US (179). Since MPS I and II have the relatively highest incidences at birth, compared to the other types of MPS, we will mainly focus on these two types of diseases. In addition, we will present types of MPS VI and VII, which have shown a link with cancer development, even though they have very low incidences at birth.

MPS I is an autosomal recessive disorder characterized by alpha-L-iduronidase (IDUA) enzyme deficiency, which is caused by a mutation in the *IDUA* gene, leading to the accumulation of dermatan sulfate and heparan sulfate in several organs and tissues. MPS I can show various degrees of clinical manifestations, and therefore is categorized according to its severity: the most severe form of MSP I is Hurler syndrome, the moderate form is Hurler-Scheie, and the least severe is Scheie syndrome (177). Currently, there is limited knowledge about the involvement of IDUA in cancer. One study found that tumors from breast cancer patients with visceral metastasis had significantly decreased IDUA expression levels compared to those without visceral metastasis. Suggesting an association between IDUA gene expression with the development of visceral organ metastasis and survival of breast cancer patients (107). Another study by Liu et al. performed a bioinformatics analysis with data from the Gene Expression Omnibus (GEO) database and obtained a glycometabolism-related gene set associated with the overall survival of patients with ovarian cancer. They were able to identify IDUA as a prognostic gene of ovarian cancer. In addition, they analyzed the expression of IDUA in ovarian cancer cells. Results showed that IDUA expression was significantly downregulated compared to human ovarian epithelial cells (108). However, additional studies are needed to elucidate the role and understand the mechanistic relationship between IDUA and cancer.

MPS II, also known as Hunter Syndrome, is caused by an inherited mutation in the *IDS* gene encoding for the iduronate-2-sulfatase (IDS) enzyme, resulting in dermatan sulfate and heparan sulfate accumulation. Presently, there is one research study exploring the potential relationship of IDS with cancer. Singh et al. found depleted IDS levels in invasive malignant epithelia of breast cancer sections compared to non-invasive or untransformed breast tissues. Simultaneously, there was a rise in levels of dermatan sulfate in the extracellular environment. Following a reduction in IDS levels, non-invasive breast cancer (MCF-7) cells displayed an increase in invasion and a shift towards a mesenchymal morphology with cytoplasmic protrusions on collagen matrices, whereas control cells retained their polygonal shape. These findings suggest that transformed cells may secrete dermatan sulfate, which can modify the mechanical characteristics and polymeric organization of nearby collagen fibers. This, in turn, may promote improved interaction between cells and the ECM, and facilitate mesenchymal migration of breast cancer cells (109).

Furthermore, it should be noted that other types of MPS, such as Type VI (Maroteaux-Lamy syndrome) caused by mutations in the *ARSB* gene, leading to deficiency of arylsulfatase B (ARSB; also known as N−acetylgalactosamine−4−sulfatase), has also been found to be associated with cancer development. The main role of ARSB is to break down GAGs into dermatan sulfate and chondroitin sulfate (180). In melanoma cells, ARSB activity was decreased compared to normal melanocytes. The decrease of ARSB activity resulted in the overexpression of melanoma progression factors, such as chondroitin sulfate proteoglycan 4 (CSPG4) and pro-matrix metalloproteinase 2 (pro-MMP2), causing increased invasiveness of melanoma cells (110). A decrease of ARSB activity in colorectal cancer cells compared to colonic epithelial cells, demonstrated an increase in cell adhesion, migration, and invasion, through upregulation of MMP9 expression and RhoA activation, which are mediators of cellular motility, implicating a key role of ARSB activity in the metastatic potential of epithelial cells (111). In addition, a decline in ARSB activity has been shown in prostate and breast carcinoma cells, which is associated with an increase in total sulfated GAGs and chondroitin sulfate content in malignant cells, suggesting a role in cell-to-cell and cell-to-matrix interactions (112, 113).

MPS Type VII (Sly syndrome) caused by β−glucuronidase deficiency involving the *GUSB* gene, has also been linked to cancer development. Several studies have reported that β−glucuronidase activity was higher in different cancers, such as highly invasive colorectal carcinoma cells compared to poorly invasive cells (114), gastric cancer compared to non-cancerous tissues (115), and pancreatic cancer due to an increased steady-state level of the enzyme compared to healthy pancreas (116). These results suggest that increased β-glucuronidase is closely related to tumor progression and metastasis. Moreover, a recent article investigating the resistance mechanism of anti-PD1 (programmed cell death 1 protein) found that *GUSB* expression was higher in HCC tumors that do not respond to anti-PD1 treatment compared to responding tumors. Anti-PD1 therapy has been shown to play a major role in inhibiting effector immune cell depletion, resulting in successful treatment advances (181, 182). However, HCC tumors can develop resistance against anti-PD1 (183). It was found that increased *GUSB* expression in HCC cells promotes cancer cell growth, reduced PD-L1 expression, and immunosuppression. In contrast, silencing *GUSB* prevents proliferation, invasion, and migration of HCC human cells, upregulation of PD-L1 expression, increased NK and T-cells in the tumor microenvironment, and decreases immunosuppressive cells such as regulatory T-cells (Tregs) and M2 macrophages. Therefore, inhibiting *GUSB* expression offers a novel strategy to reduce HCC cell progression and improve the sensitivity to anti-PD1 therapy (117).

## 5 Fatty acid oxidation disorders

**Figure 3:**
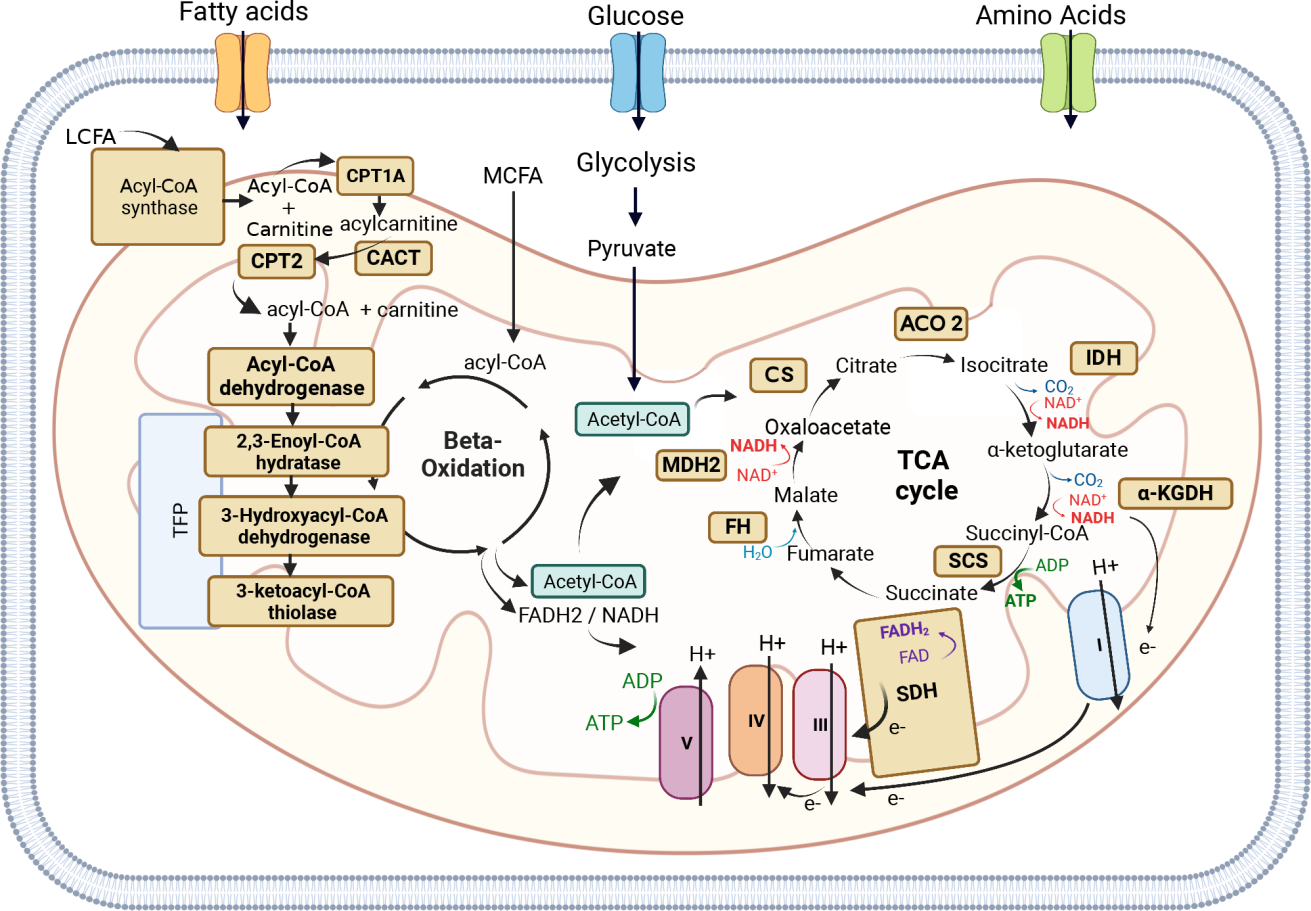
Mitochondrial Fatty Acid Beta Oxidation and TCA cycle. Mitochondrial Fatty Acid Beta Oxidation (FAO) is a major multi-step process by which cells break down fatty acids and catabolize them into acetyl-coA, which subsequently enters the tricarboxylic acid (TCA). The FADH2 and NADH produced by FAO are used by the Electron Transport Chain (ETC) to produce ATP. Long-chain fatty acids (LCFA) cannot enter mitochondria through passive diffusion, like Medium-chain fatty acids (MCFAD), and need to be activated into fatty acyl-coenzyme A in the cytosol by acetyl-CoA synthetase, and then conjugated to carnitine to be imported into the mitochondrial matrix. The shuttle of LCFA into mitochondria is carried out by three enzymes: the outer mitochondrial membrane enzyme Carnitine palmitoyltransferase 1A (CPT1A), the mitochondrial intermembrane space enzyme Carnitine-acylcarnitine translocase (CACT), and the inner mitochondrial membrane enzyme Carnitine palmitoyltransferase 2 (CPT2). Once fatty acyl-CoA is inside the mitochondrial matrix, it can enter the beta-oxidation cycle to produce acetyl-CoA, which can subsequently enter the TCA cycle. Four main enzymes are involved in the beta-oxidation cycle: acyl-CoA dehydrogenase, 2,3-Enoyl-CoA hydratase, 3-Hydroxyacyl-CoA dehydrogenase, and 3-Ketoacyl-CoA thiolase. The other beta-oxidation steps are catalyzed by the mitochondrial trifunctional protein (TFP or MTP), a protein complex attached to the inner mitochondrial membrane composed of two types of subunits: the alpha subunit (TFPα) and the beta subunit (TFPβ). The TFPα subunit comprises the 2,3-enoyl-CoA hydratase and 3-hydroxyacyl- CoA dehydrogenase activities, whereas the TFPβ subunit comprises the 3-Ketoacyl-CoA thiolase activity. The TCA cycle is a key metabolic node whose main function is to generate electrons to fuel the mitochondrial ETC (mETC) for ATP production. The breakdown of fatty acids (beta-oxidation), glucose (glycolysis), and some amino acids leads to the production of Acetyl-CoA, which can then enter the TCA cycle. Acetyl-CoA is a key substrate that participates in the first reaction of the TCA cycle, ensured by Citrate Synthase (CS) enzyme which converts oxaloacetate into citrate. The second reaction of the TCA cycle leads to the conversion of citrate into isocitrate by Aconitase (ACO2), which converts citrate into isocitrate. Isocitrate is then converted by Isocitrate Dehydrogenase (IDH), during the third reaction of the TCA cycle into α-ketoglutarate. α-ketoglutarate is converted into Succinyl-CoA by α-Ketoglutarate Dehydrogenase (α-KGDH). Succinyl-CoA is then converted by Succinyl-CoA synthetase (SCS) into succinate, which is then converted by Succinate dehydrogenase (SDH or mETC Complex II) into fumarate. Fumarate is converted into malate by Fumarate Hydratase (FH). The last reaction of the cycle is the conversion of malate into oxaloacetate by Malate Dehydrogenase (MDH2). The mETC is composed of 5 enzymatic complexes: Complex I-V. Electrons generated by the TCA cycle funnel through the mETC allowing the complexes I, III, and IV to pump protons generating a membrane potential used by the complex V to generate ATP. The Figure was partly generated using Biorender under the agreement number: VS25LLE9OH (www.Biorender.com).

Mitochondrial Fatty Acid Beta Oxidation (FAO) is a major multi-step process by which cells break down fatty acids and catabolize them into acetyl-coA, which subsequently enters the tricarboxylic acid (TCA) cycle resulting in the production of more ATP than the oxidation of carbohydrates (**Figure 3**) (184). Beta-oxidation is an important source of energy, especially during periods of high-energy demand such as fasting or exercise, but also for high-energy dependent tissues, such as the heart, muscle, liver, and brain. This is why mutations in the genes coding for the enzymes involved in either the beta-oxidation cycle or the transport of long-chain fatty acids (LCFA) into mitochondria can lead to severe inherited metabolic FAO disorders (FAODs) (185, 186). Unlike Medium or Short-chain fatty acids, LCFA cannot enter mitochondria through passive diffusion and need to be activated into fatty acyl-coenzyme A in the cytosol by acetyl-CoA synthetase, and then conjugated to carnitine to be imported into the mitochondrial matrix (187).

The shuttle of LCFA into mitochondria is carried out by three enzymes: the outer mitochondrial membrane enzyme Carnitine palmitoyltransferase 1A (CPT1A), the mitochondrial intermembrane space enzyme Carnitine-acylcarnitine translocase (CACT), and the inner mitochondrial membrane enzyme Carnitine palmitoyltransferase 2 (CPT2). CPT1 catalyzes the rate-limiting step of FAO by converting fatty acyl-CoA into acyl-carnitine, which is then transported into the mitochondrial matrix via CACT. CPT2 carries out the last reaction by converting carnitine into Acyl-CoA, which can then enter the beta-oxidation cycle (**Figure 3**) (185, 186, 188, 189). Alterations in either of the three enzymes (CPT1A, CACT, and CPT2) can prevent the body from using certain types of fatty acids leading to hypoketotic hypoglycemia (decreased glucose in the blood) under fasting conditions or during exercise. Moreover, CPT2 deficiency has more severe clinical presentations than CPT1 deficiency (189). It is interesting to note that while CPT1A and CPT2 are involved in the same metabolic pathway, their levels of expression, such as CPT1A upregulation and CPT2 downregulation, can have opposite effects in different types of cancer. CPT1A upregulation has been found to promote the proliferation, survival, and invasion of several cancer types, including colorectal cancer (190–192), nasopharyngeal cancer (193), ovarian cancer (194, 195), glioblastoma (196), gastric cancer (197), and HCC (198), and in many cases is associated with poor prognosis and metastasis. In contrast, downregulation of CPT2 was found to be associated with poor prognosis and tumorigenesis in colorectal cancer (199–201) and HCC (198, 202).

The elevated expression of CPT1A has been observed in metastatic tumors compared to primary tumors of colorectal cancer patients (191). Wang et al. showed that CPT1A upregulation promotes metastasis of detached colorectal cancer cells by inhibiting anoikis, a programmed cell death that occurs when cells detach from the ECM, while a decrease in metastasis was observed in CPT1A-depleted colorectal cancer cells (190). An *in vitro* study showed that adipocytes co-cultured with colon cancer cells release fatty acids which are taken up by cancer cells, allowing them to survive in nutrient-deprived conditions by upregulating mitochondrial FAO. Whereas *in vivo* studies showed that co-injection of adipocytes with colon cancer cells promotes tumor growth (191), silencing CPT1A in colon cancer cells eliminated the protective effect of fatty acids against nutrient deprivation and decreased the expression of genes associated with cancer stem cells downstream of the Wnt/β-catenin pathway (192). This suggests that the presence of adipocytes in the TME are a source of energy and metabolic regulators, facilitating the survival and proliferation of colon cancer cells. Additionally, CPT1A upregulation has been observed in radiation-resistant nasopharyngeal cancer cells associated with Rab14 (a GTPase), which facilitates fatty acid trafficking from lipid droplets to the mitochondria where FAO takes place, resulting in decreased radiation-induced lipid accumulation, demonstrating a role for CPT1A in radiation resistance (193). Moreover, CPT1A is overexpressed in most ovarian cancer cell lines, primary ovarian serous carcinomas, and a subset of high-grade serous ovarian cancers (HGSOCs) (194, 195). Studies *in vitro* showed CPT1A deficiency in ovarian cancer cell lines results in decreased cellular ATP levels, cell cycle arrest, suppression of anchorage-independent growth, and reduced xenograft formation through the induction of p21 (cyclin-dependent kinase inhibitor) by activation of the transcription factor FoxO by AMPK, JNK, and p38 (194).

On the other hand, the downregulation of CPT2 in colorectal cancer promotes cell proliferation capacity (199, 200) and inhibits apoptosis by decreasing p53 expression (200). In addition, the downregulation of CPT2 in colorectal cancer can promote cancer stemness and oxaliplatin (chemotherapy drug) resistance through the activation of the Wnt/β-catenin pathway by inducing glycolytic metabolism (201). In HCC tissues and serum of HCC patients, the accumulation of acylcarnitines, which serve as carriers to transport activated LCFA into the mitochondria for beta-oxidation, could be attributed to CPT2 downregulation, leading to the suppression of beta-oxidation and metabolic reprogramming to escape lipotoxicity and promote hepatocarcinogenesis (198). Moreover, the downregulation of CPT2 has been shown to have a link to human nonalcoholic fatty liver disease (NAFLD)-related hepatocarcinogenesis. Elevated levels of transcription factors E2F1 and E2F2 were observed in NAFLD, suggesting that these transcription factors may be metabolic drivers of HCC by promoting a lipid-rich environment (203). In glioblastoma multiforme, enhanced fatty acid metabolism by co-enhancement of CPT1A and CPT2 and immune checkpoint CD47, which functions as an anti-phagocytic signal, promotes the growth of radioresistant glioblastoma multiforme cells. By blocking FAO there is a reduction of CD47 anti-phagocytosis and tumor growth. Targeting the FAO-CD47 axis could therefore be an efficient way to block the growth of radioresistant glioblastoma multiforme cells (196).

Once fatty acyl-CoA is inside the mitochondrial matrix, it can enter the beta-oxidation cycle to produce acetyl-CoA, which can subsequently enter the TCA cycle. Four main enzymes are involved in the beta-oxidation cycle: acyl-CoA dehydrogenase, 2,3-Enoyl-CoA hydratase, 3-Hydroxyacyl-CoA dehydrogenase, and 3-Ketoacyl-CoA thiolase (also known as acetyl-CoA transferase) (**Figure 3**). The beta-oxidation cycle can be described in four steps: (i) Fatty acyl-CoA is dehydrogenated by acetyl-CoA dehydrogenase resulting in the formation of 2,3-enoyl-CoA, (ii) 2,3-enoyl-CoA is hydrated to form 3-hydroxyacyl-CoA by 2-enoyl-CoA hydratase, (iii) 3-hydroxyacyl-CoA is dehydrogenated by 3-hydroxyacyl-CoA dehydrogenase to form the 3-ketoacyl-CoA compound, and (iv) 3-ketoacyl-CoA is cleaved by thiolase yielding acetyl-CoA and an acyl-CoA two carbons shorter than the original, which can re-enter at the first step in the beta-oxidation pathway.

The first beta-oxidation step is catalyzed by various acyl-CoA dehydrogenases, each with a specific affinity towards different fatty acyl chain lengths. Acyl-CoA Dehydrogenase Very-Long Chain (ACADVL or VLCAD) and Acyl-CoA Dehydrogenase Medium-chain (ACADM or MCAD), are two types of Acyl-CoA dehydrogenases that initiate beta-oxidation of Very-Long Chain Acyl-CoA esters and Medium-Chain Acyl-CoA esters, respectively. Deficiencies in these enzymes are common in FAOD and result in hypoketotic hypoglycemia, liver dysfunction, and liver failure. VLCAD deficiency is clinically distinct, causing rhabdomyolysis (muscle tissue breakdown releasing myoglobin) and cardiomyopathy, which are not observed in MCAD deficiency (204). Recent studies have shown that the downregulation of VLCAD in human HCC tissues and cells promotes cell proliferation and metastasis (205). On the other hand, in glioblastoma, MCAD plays a crucial role in protecting cancer cell integrity against the accumulation of toxic by-products that would otherwise affect mitochondrial activity, demonstrating the non-energetic role of FAO enzymes in the dependence on fatty acid metabolism in cancer (205, 206).

The other three beta-oxidation steps are catalyzed by the mitochondrial trifunctional protein (TFP or MTP), a protein complex attached to the inner mitochondrial membrane (207, 208). TFP is composed of two types of subunits: the alpha subunit (TFPα), encoded by the *HADHA* gene, and the beta subunit (TFPβ), encoded by the *HADHB* gene. The TFPα subunit comprises the 2,3-enoyl-CoA hydratase and 3-hydroxyacyl-CoA dehydrogenase activities, whereas the TFPβ subunit comprises the 3-Ketoacyl-CoA thiolase activity (**Figure 3**) (209). Mutations of *HADHA* or *HADHB* genes leads to TFP deficiency, an autosomal recessive disorder affecting LCFA oxidation characterized by hypoglycemia, hypotonia (decreased muscle tone), and liver dysfunction (210, 211). The TFP is a promising target to restrain tumor growth in lung carcinomas by targeting the activity of the *HADHA* enzyme (212, 213). Ameodo et al. observed a metabolic heterogeneity between human biopsies of lung adenocarcinomas and divided them into two subgroups: (i) tumors with a low mitochondrial respiration and (ii) tumors with a high mitochondrial respiration. This second group was poorly relying on glucose and was presenting an increased expression of the TFP enzyme *HADHA* compare to the adjacent tissue. Inhibition of the TPF activity *in vivo* leads to a reduction of tumor growth (212). Moreover, both *HADHA* and *HADHB* enzymes have been found overexpressed in malignant lymphoma progression (214, 215), relying on fatty acid metabolism and notably FAO as a key metabolic pathway for tumor progression and survival (216, 217). Additionally, in colorectal cancer and stomach adenocarcinoma, *HADHB* has been proposed as a tumor suppressor, its expression being significantly lower in tumors compared to normal tissue (218, 219).

Glucose and amino acids have been well-studied in cancer metabolism and are considered important sources of energy to fuel tumor growth and survival. It is also well known that cancer cells can rely on fatty acid metabolism, and notably *de novo* lipid synthesis, an anabolic pathway, for their proliferation and survival. Over the last decade, research has highlighted how cancer cells can also rely on FAO (catabolic pathway) reshaping our view on how tumors can use lipid metabolism to their advantage (220, 221). **Table 4** encompasses details about mutated enzyme names, enzymatic roles, diseases linked to the enzymes, and the regulation (up or down) of these enzymes in different cancer types involved in Fatty Acid Oxidation. Further research is needed to fully characterize the energetic and non-energetic roles that FAO enzymes can play to promote cancer progression.

**Table 4:** Fatty Acid Oxidation Disorders (FAODs).

Enzymes	Role	Disease Name	Upregulated Cancers	Downregulated Cancers
Carnitine palmitoyltransferase 1A (CPT1A)	Catalyzes the transfer of the acyl group of a long-chain fatty acyl-CoA from coenzyme A to carnitine.	Carnitine palmitoyltransferase I (CPT I) deficiency or CPT 1A deficiency	Colorectal cancer (190–192), nasopharyngeal cancer (193), ovarian cancer (194, 195), glioblastoma (196), gastric cancer (197), and HCC (198).	
Carnitine palmitoyltransferase 2 (CPT2)	Catalyzes the re-conjugation of long and very-long-chain acyl-carnitines to acyl-CoA	Carnitine palmitoyltransferase II (CPT II) deficiency or CPT2 deficiency		Colorectal cancer (199–201) and HCC (198, 202).
Acyl-CoA Dehydrogenase Very-Long Chain (ACADVL or VLCAD)	Breaks down a group of very long-chain fatty acids	Very long-chain acyl-CoA dehydrogenase (VLCAD) deficiency		HCC (205).
Acyl-CoA Dehydrogenase Medium-chain (ACADM or MCAD)	Breaks down a group of medium-chain fatty acids.	Medium-chain acyl-CoA dehydrogenase (MCAD) deficiency	Glioblastoma (205, 206).	
Mitochondrial trifunctional protein (TFP or MTP), composed of two types of subunits: the alpha subunit (TFPα; *HADHA* gene), and the beta subunit (TFPβ; *HADHB* gene).	Catalyzes the last three reactions in the fatty acid β-oxidation process. Breaks down long-chain fatty acids.	Mitochondrial trifunctional protein deficiency or MTP deficiency	*HADHA* in lung carcinomas (212, 213) and both *HADHA* and *HADHB* in malignant lymphoma (214, 215).	*HADHB* in colorectal cancer and stomach adenocarcinoma (218, 219).

## 6 Mitochondrial disorders

The most common inherited metabolic disorders are mitochondrial disorders caused by dysfunction of mitochondrial activity (222). The mitochondria is a key cellular organelle, known as the powerhouse of the cell, which ensures energy production in the form of ATP. The mitochondrial machinery relies on genes from both nuclear DNA (nDNA) and mitochondrial DNA (mtDNA). The mtDNA codes for 2 rRNAs, 22 tRNAs, and 13 proteins, which are part of the multi-subunit enzymatic complexes of the electron respiratory chain (ETC) (223, 224). The TCA cycle, also known as the Krebs cycle, is a key metabolic node whose main function is to generate electrons to fuel the ETC for ATP production (225). The TCA cycle comprises 8 enzymes, all encoded by genes located in the nDNA. Electrons generated by the TCA cycle allow the ETC to generate a membrane potential, which is used to convert ADP into ATP, a process called oxidative phosphorylation (OXPHOS) (**Figure 3**). Mutations in genes encoding the enzymes involved in the TCA cycle and OXPHOS can lead to mitochondrial disorders and cancer, due to the inability of mitochondria to produce energy. **Table 5** encompasses details about enzyme mutations involved in the TCA cycle, their respective enzymatic roles, associated diseases, and the regulation status (up or down) of the enzymes and genes across diverse cancer types.

**Table 5:** Mitochondria Disorders – TCA Cycle.

Enzymes	Role	Disease Name	Upregulated Cancers	Downregulated Cancers
Citrate synthase (CS)	Binds with the oxaloacetate and reacts with acetyl-CoA, leading to the production of citrate.		Ovarian (226), pancreas (227), and colon (228).	Cervical (229).
Aconitase (ACO2)	Catalyzes the conversion of citrate into isocitrate	Cerebellar-retinal degeneration (230, 231) and with severe optic atrophy and spastic paraplegia (232).	HCC (233).	Gastric cancer (234) and colorectal cancer (235).
Isocitrate dehydrogenase (IDH). Three IDH isoforms exist IDH1, IDH2, and IDH3.	Converts of isocitrate into α-ketoglutarate (α-KG)		*IDH1* and *IDH2* in glioblastoma (236) *IDH2* in colorectal (237) and lung (238). IDH3-a in glioblastoma (239) and in HCC (240).	
α-ketoglutarate dehydrogenase (α-KGDH), also called 2-oxoglutarate dehydrogenase (OGDH).	Converts α-KG into succinyl-CoA	alpha-ketoglutarate dehydrogenase complex (KGDHC) deficiency	Gastric (241).	
Succinyl-CoA synthetase (SCS), also known as Succinyl-CoA ligase. SCS is composed of two subunits, an α-subunit which is encoded by the gene *SUCLG1*, and the β-subunit which is encoded by the gene *SUCLA2* (specificity for ADP), or by the gene *SUCLG2* (specificity for GDP).	Breaks down succinyl-CoA into succinate and free CoA, and converts ADP or GDP into ATP or GTP, respectively.	Succinyl-CoA ligase deficiency	*SUCLG1* in acute myeloid leukemia (242).	*SUCLA2* in prostate (243).
Succinate dehydrogenase (SDH), also known as Succinate-coenzyme Q reductase (SQR). SDH is composed of four subunits, *SDHA*, *SDHB*, *SDHC* and *SDHD*.	Catalyzes the oxidation of succinate to fumarate and transfers electrons from succinate to ubiquinone (coenzyme Q).	Succinate dehydrogenase (SDH) deficiency		*SDHB* in ovarian (244).
Fumarase, also known as fumarate hydratase	Catalyzes the hydration of fumarate into L-malate.	Fumarase deficiency, also known as Fumarate hydratase deficiency or Fumaric aciduria.		Clear cell renal carcinomas (245).
Malate dehydrogenase (MDH2)	Catalyzes the reversible conversion of malate into oxaloacetate	Mitochondrial malate dehydrogenase (MDH2) deficiency	Prostate (246).	

### 6.1 Tricarboxylic acid cycle

The first reaction of the TCA cycle is catalyzed by citrate synthase (CS), which binds with the oxaloacetate and reacts with acetyl-CoA, leading to the production of citrate. Chen et al. found that CS was upregulated in human ovarian tumors and human ovarian tumor cell lines. Knockdown of CS in ovarian cancer cells leads to decreased cell proliferation accompanied by downregulation of ERK phosphorylation, inhibition of cell migration and invasion with decreased expression of p-FAK, MMP2, and Vimentin, and decreased drug resistance by downregulation of *ATG12* (226). Additionally, the activity of CS is significantly higher in human pancreatic ductal carcinoma compared with adjacent nonneoplastic tissue, contributing to the conversion of glucose to lipids, which provides the substrate for membrane lipid synthesis in pancreatic cancer (227). In colon cancer cells, CS has been shown to interact with SIRT5, a nicotinamide adenine dinucleotide (NAD)^+^-dependent deacetylase. SIRT5 dessucinylates CS, regulating its enzymatic activity, whereas hypersuccinylation of CS reduces its enzymatic activity and inhibits the proliferation and migration of colon cancer cells (228). Furthermore, Lin et al. found that reduced expression of CS in human cervical cancer cells leads to a change in cellular energy production, from mitochondrial aerobic respiration to cytosolic glycolysis. This change is accompanied by the induction of EMT, which results in accelerated tumor malignancy due to the deregulation of p53 functions and abnormal cell growth signaling (229).

The second reaction of the TCA cycle is ensured by an aconitase (ACO2) which catalyzes the conversion of citrate into isocitrate. ACO2 is a key enzyme of the TCA cycle and is also involved in lipid metabolism. Citrate can be exported from the mitochondrial matrix to the cytosol to be converted back into oxaloacetate and acetyl-CoA, which can be used for fatty acid synthesis. The reduced ACO2 enzyme activity in cells can lead to a deficiency in cellular respiration, mitochondrial DNA depletion, and altered expression of some TCA components and electron transport chain subunits (247). ACO2 mutations have been associated with cerebellar-retinal degeneration (230, 231) and with severe optic atrophy and spastic paraplegia (232). ACO2 expression has been found dysregulated in different types of cancers and linked to tumor progression. Decreased expression of ACO2 is associated with poor prognosis in gastric cancer (234) and colorectal cancer (235) by promoting a switch from mitochondrial oxidative phosphorylation to glycolysis in the cytosol. The knockdown of ACO2 in colorectal cancer promotes cell proliferation and colorectal cancer growth (235). However, compared with normal hepatocytes, ACO2 was overexpressed in HCC cells, promoting cell proliferation and migration by affecting molecular pathways involved in cellular energy metabolism, metabolite changes, and fatty acid metabolic pathway (233).

The third step of the TCA cycle is the conversion of isocitrate into α-ketoglutarate (α-KG) by the enzyme isocitrate dehydrogenase (IDH). Three IDH isoforms exist IDH1, IDH2, and IDH3. IDH1 is present in the cytosol and the peroxisome, while IDH2 and IDH3 are present in the mitochondrial matrix. IDH1 and IDH2 are both NADP^+^-dependent homodimers and catalyze the reversible conversion of isocitrate into α-KG. By contrast, IDH3 is an NAD^+^-dependent heterotetrameric protein composed of two α subunits (*IDH3A*), one β subunit (*IDH3B*), and one γ subunit (*IDH3G*), that catalyzes the irreversible conversion of isocitrate into α-KG (248). The α subunit ensures the catalytic activity of the holoenzyme, requiring the function of the β and γ subunits (249). IDH2 and IDH3 are both involved in the TCA cycle. The IDH2 catalytic activity results in the reduction of NAPD^+^ into NAPDH, and the IDH3 catalytic activity results in the production of the electron donor NADH. *IDH1* and *IDH2* are the most frequently mutated metabolic genes in human cancer, and their mutations have been identified in different types of cancer, notably in gliomas, secondary glioblastomas, cartilaginous and bone tumors, and acute myeloid leukemia (236, 250–252). Mutated *IDH1* and *IDH2* acquire a new ability by converting α-KG into the oncometabolite 2-HG (2-hydroxyglutarate), accumulation of which can lead to the modification of the epigenome, notably by inhibiting α-KG-dependent dioxygenases (248, 252–254). While the role of the mutant IDH2 in cancer has been well characterized, recent studies have shown there is a pro-tumorigenic role for wild-type IDH2 as well. In colorectal cancer, wild-type IDH2 protects cancer cells against ROS-mediated DNA damage (237). Additionally, in lung cancer cells, the overexpression of IDH2 decreases α-KG concentrations, enhances the production of 2-HG, and decreases ROS levels, protecting cancer cells against DNA damage. The downregulation of α-KG promotes the transcription of HIF1α-targeted glycolytic genes (238). While mutated IDH1 and IDH2 are cancer-driver genes through the production of 2-HG and its impact on the epigenome, IDH3 has not been characterized as such in cancer. A study showed that IDH3-a is elevated in glioblastoma, and loss of function decreases TCA cycle turnover and inhibits oxidative phosphorylation (239). Moreover, IDH3-a is upregulated in HCC tissues and is associated with increased tumor size and greater clinicopathologic stage of HCC. *In vitro* studies showed that IDH3-a promotes EMT by increasing metastasis associated 1 (MTA1), an oncogene involved in the progression of cancer cells to metastasis, thereby enabling migration and invasion of HCC cells (240).

The fourth reaction of the TCA cycle is the conversion of α-KG into succinyl-CoA, leading to the reduction of NAD^+^ into NADH, an electron donor which directly transfers electrons to complex I of the ETC. This reaction is catalyzed by α-ketoglutarate dehydrogenase (α-KGDH), also called 2-oxoglutarate dehydrogenase (OGDH), a highly regulated enzyme, whose role in carcinogenesis has been unclear until recently (255). The levels of OGDH in gastric cancer tissues are highly upregulated compared to normal tissues, which correlates with poor clinicopathological parameters for gastric cancer patients. The overexpression of OGDH results in decreased EMT epithelial markers, mitochondrial membrane potential, oxygen consumption rate, intracellular ATP product, and upregulation of EMT mesenchymal markers, ROS levels, and NADP^+^/NAPDH ratio, and facilitated the activation of Wnt/β-catenin signal pathway. In addition, the overexpression of OGDH promoted tumorigenesis of gastric cancer cells in nude mice (241).

The fifth reaction of the TCA cycle is catalyzed by Succinyl-CoA synthetase (SCS; also known as succinate-CoA ligase), which breaks down succinyl-CoA into succinate plus free CoA, and converts ADP or GDP into ATP or GTP, respectively. SCS is composed of two subunits, an α-subunit which is encoded by the gene *SUCLG1*, and the β-subunit which is encoded by the gene *SUCLA2* (specificity for ADP), or by the gene *SUCLG2* (specificity for GDP) (256). Mutations in both *SUCLG1* and *SUCLA2* have been associated with encephalomyopathic mtDNA depletion syndrome with methylmalonic aciduria (257). *SUCLG1* mutations can lead to severe lactic acidosis and elevated levels of methylmalonic acid and pyruvic acid in the blood and urine. While, *SUCLA2* mutations can lead to hypotonia (decreased muscle tone), muscle weakness, Leigh syndrome (a severe neurological disorder), dystonia (movement disorder), and sensorineural hearing loss (256). Increased *SUCLG1* expression in acute myeloid leukemia patients is associated with a decreased percent survival and identifies as a risky prognostic gene (242). *SUCLA2* has been previously shown to be significantly downregulated in prostate cancer (243). A model presented by Wang et al. predicts that in malignant prostate cancer cells, the GTP-specific beta subunit of succinyl-CoA synthetase (*SUCLG2*) is selectively lethal because the alternative route via ATP-specific succinyl-CoA synthetase (*SUCLA2*) is not present in these cells, creating a selective vulnerability to *SUCLG2* knockdown in malignant cells (258). Additionally, a recent study found that the overexpression of the epidermal growth factor receptor (EGFR) in prostate cancer cells leads to the upregulation of the ligand for the LIF receptor (LIFR). The upregulation of LIFR in turn leads to the overexpression of *SUCGL2*, an enzyme involved in the production of succinate. The increased production of succinate promotes the neuroendocrine differentiation of prostate cancer cells, which makes them more resistant to androgen deprivation therapy (ADT) (259).

The sixth reaction of the TCA cycle is ensured by Succinate dehydrogenase (SDH), also called Succinate-coenzyme Q reductase (SQR), a mitochondrial metabolic enzyme complex (respiratory complex II) involved in both the TCA cycle and OXPHOS. SDH catalyzes the oxidation of succinate to fumarate and then transfers electrons from succinate to the ubiquinone pool of the ETC via the electron donor FADH2 (260–262). SDH is composed of four subunits, *SDHA* and *SDHB* subunits that ensure the catalytic activity of the SDH complex, and *SDHC* and *SDHD* subunits that anchor the complex to the inner mitochondrial membrane (263, 264). The subunits of this complex are exclusively encoded by genes located in the nDNA (260, 265). Mutations have been identified in the genes *SDHA*, *SDHB*, and *SDHD* and in one assembly gene factor (*SDHAF1*) in patients presenting Complex II deficiency (266, 267). Moreover, germline mutations of *SDHB*, *SDHC*, or *SDHD*, are associated with an increased risk of aggressive variants of renal cell carcinoma (264, 268–270). In addition, *SDHB* was found to be decreased in ovarian tumors. The knockdown of *SDHB* in mouse ovarian cancer cells increases proliferation, promotes EMT, and leads to histone hypermethylation. In *SDHB*-depleted cells, the amount of glucose fueling the TCA cycle is decreased and is compensated by an increase of glutamine, a contribution to sustaining TCA cycle activity. This suggests that the glucose entering the pentose phosphate pathway is increased in *SDHB*-deficient cells to sustain nucleotide biosynthesis and rapid proliferation (244).

The seventh step of the TCA cycle is ensured by fumarase or fumarate hydratase (encoded by the gene *FH*), which catalyzes the conversion of fumarate into malate. *FH* deficiency results in neonatal and infantile encephalopathy (271–273). Germline mutations of *FH* are associated with Multiple Cutaneous Leiomyomas with Uterine Leiomyomas (MCUL) syndrome, also known as Reed syndrome, and share features with hereditary leiomyomatosis and renal cancer cell (HLRCC) (274–276). HLRCC is a hereditary condition that causes the development of multiple leiomyomas (fibroids) in the skin and uterus, and an increased risk of developing renal cell carcinoma (277). Individuals with hemizygous germline *FH* mutations have an increased risk of renal cancer. The remaining wild-type allele in these tumors is often functionally inactivated, suggesting that FH inactivation promotes tumor development. The study shows that *FH* inhibition and the resulting elevation of intracellular fumarate leads to the upregulation of hypoxia-inducible factors (HIFs), which are involved in many cancers including clear cell renal carcinomas (245). In addition, an aggressive subtype of renal cell carcinoma caused by mutations in the *FH* gene is Fumarate hydratase (FH)-deficient renal cell carcinoma (FHdRCC), which can lead to fumarate accumulation, resulting in the activation of HIF through the inhibition of prolyl hydroxylases. HIF activation promotes tumorigenesis by inducing a metabolic shift to glycolysis, promoting the transcription of genes such as vascular endothelial growth factor (VEGF), and a tumor-promoting mechanism between HIF and EGFR (278).

The eighth and last reaction of the TCA cycle is ensured by Malate Dehydrogenase (MDH2), which catalyzes the reversible conversion of malate into oxaloacetate. MDH2 deficiency has been shown recently to lead to early-onset severe encephalopathy, a cause of Leigh syndrome, and has been identified as a pheochromocytoma and paraganglioma susceptibility gene (279, 280). Moreover, the overexpression of the gene MDH2 was associated with shorter relapse-free survival in prostate cancer patients who underwent chemotherapy. The knockdown of MDH2 in prostate cancer cell lines decreased cell proliferation, increased sensitivity to the chemotherapy drug docetaxel, and affected signaling pathways and metabolic efficiency by influencing JNK signaling and oxidative metabolism (246).

### 6.2 Oxidative phosphorylation

Glycolysis and FAO fuel the TCA cycle which transfers electrons to the ETC to generate ATP through OXPHOS. The ETC is composed of 5 enzymatic multi-subunit complexes (CI-CV) (**Figure 3**). Complex I, also known as NADH dehydrogenase, plays a crucial role by facilitating the oxidation of NADH to NAD+. Complex II, also known as succinate dehydrogenase, facilitates the conversion of succinate to fumarate through oxidation. Complex III, commonly known as Cytochrome c reductase, has the pivotal function of reducing cytochrome c. Complex IV, known as Cytochrome c oxidase, has a crucial function in the oxidation of cytochrome c. Finally, Complex V, commonly referred to as ATP synthase, earns its name from its essential role in the synthesis of ATP utilizing the proton motive force (281). Electrons go through a series of redox reactions when passing through the ETC complexes CI, CIII, and CIV releasing energy used by the complexes CI, CIII, and CIV to pump protons (H^+^) from the mitochondrial matrix resulting in the generation of a membrane potential. This membrane potential is then used by Complex V to catalyze the conversion of ADP and inorganic phosphate into ATP (282). Mutations in genes involved in the respiratory chain complex biogenesis or activity leads to mitochondrial diseases, notably Leigh syndrome, MELAS (mitochondrial encephalopathy, lactic acidosis and stroke-like episodes) syndrome, MERRF (myoclonic epilepsy with ragged red fibers (MERRF) syndrome, and mitochondrial myopathies (283).

As cancer cells rewire their metabolism to use glucose through aerobic glycolysis, one of the causes could be mitochondrial defects (284). However, it has been shown that dysfunctional OXPHOS can also promote the dependence of cancer cells for aerobic glycolysis (285–287). Recent studies have highlighted that cancer cells can be highly reliant on OXPHOS for their proliferation and survival, and that the mitochondrial ETC can play an essential role in tumor growth (288–292). Birsoy et al. showed that cancer cells sensitive to low glucose levels harbor glucose use deficiencies or Complex I mutations that lead to mitochondrial dysfunction, and that these two phenomena constitute two distinct mechanisms (293). The OXPHOS pathway has also been found to be part of tumor metabolic heterogeneity. In a murine model of pancreatic ductal adenocarcinoma (PDAC), mutations of the oncogene KRAS, known to play a critical role in PDAC, lead to the death of most cancer cells but induce the survival of a subpopulation of dormant tumor cells relying on OXPHOS (294). Moreover, in PDAC (295), Acute Myeloid Leukemia (AML) (292), and triple-negative breast cancer (TNBC) (296), chemotherapy-resistant cells have been found to rely on a high OXPHOS status, while in high-grade serous ovarian cancer (HGSOC), high OXPHOS cells are chemo-sensitive (297). Metabolic heterogeneity observed in some cancers highlights the importance of combining drugs targeting different metabolic pathways to synergistically impair cancer cell proliferation and survival. Suggesting OXPHOS as a cancer vulnerability and a new potential therapeutic target (298).

Several studies have deciphered the role played by the OXPHOS complexes, specifically Complex I, for cancer cell proliferation, and the impact of their inhibition (299, 300). Mutations of genes located in both nDNA and mDNA genes coding for Complex I subunits have been found associated with Complex I deficiencies (301, 302). Complex I activity can be inhibited in cancer cells with different compounds, such as Metformin, an anti-diabetic drug, which has been investigated as a potential treatment for cancer (303, 304). Diabetic patients present increased cancer mortality compared to those without diabetes., While cancer mortality is increased when diabetic patients are treated with insulin or sulfonylureas, it is decreased when they are treated with Metformin, which slows down tumor growth (305). In human cancer cells, Metformin decreases cell proliferation in the presence of glucose and reduces hypoxic activation of HIF-1, but increases cell death upon glucose deprivation, indicating that cancer cells rely exclusively on glycolysis for survival in the presence of Metformin (306). Masoud et al. suggested that high OXPHOS cells are protected against stress induced by chemotherapy due to high mitochondrial respiration (295). Furthermore, a clinical-grade small-molecule inhibitor of Complex I, known as IACS-010759, is currently in phase I clinical trials and has been investigated in tumor growth of different types of tumors. Molina et al. has shown that IACS-010759 inhibits cell proliferation and induces apoptosis in brain cancers and AML, which are known to rely on OXPHOS, by elevating NADH levels and nucleotide monophosphates and decreasing nucleotide triphosphates (307). The inhibition of Complex I by IACS-010759 in Chronic Lymphocytic Leukemia (CLL), showed a minor effect on cell death and lead to upregulation of glucose uptake and glycolysis as a compensatory mechanism. However, the inhibition of both glycolysis and OXPHOS results in increased cell death, showing the importance of targeting multiple metabolic pathways to obtain a synergic effect (308). In addition, a study identified the therapeutic potential of targeting OXPHOS in lung tumors with SWI/SNF mutations, and demonstrated the selective anti-tumor effects of IACS-010759 in these specific tumor types (309).

Understanding the dysregulation of the TCA cycle and OXPHOS in mitochondrial disorders provides valuable insights into the pathogenesis of cancer and other related diseases. Targeting these metabolic pathways holds promise for the development of novel therapeutic strategies to fight mitochondrial disorders and improve patient outcomes.

## 7 Conclusions

The dysregulation of metabolic enzymes is intricately linked to both metabolic disorders and cancer. Metabolic reprogramming in cancer cells, characterized for a long time as the “Warburg effect,” plays a crucial role in tumorigenesis, tumor progression, and drug resistance. Understanding the dysregulation of metabolic enzymes in different metabolic pathways provides insights into the mechanisms driving these diseases. Similarities among the mechanisms described for the different groups of disorders (UCDs, GSDs, LSDs, FAODs, and mitochondrial diseases) are related to their involvement in various aspects of cellular metabolism and signaling pathways, as well as their impact on tumor growth, invasion, migration, and metastasis. Disruptions in metabolic pathways, such as pyrimidine synthesis, arginine biosynthesis, glucose metabolism, fatty acid oxidation, and mitochondrial function are some of the mechanisms that can affect energy production, nucleotide synthesis, and other essential cellular processes. In addition, several mechanisms contribute to tumor growth and proliferation, by promoting cell cycle progression, DNA synthesis, and cell division. Dysfunctional enzymes or regulators may lead to increased cell proliferation or impaired growth arrest, allowing tumors to evade normal control mechanisms and immune surveillance, leading to immunosuppression and impaired T-cell function.

Moreover, dysfunctional enzymes or metabolic alterations can impact various signaling pathways involved in tumor growth and progression. Some signaling pathways are regulated in several of the metabolic disorders, which include Wnt/β-catenin, known to regulate key cellular functions such as proliferation, differentiation, migration, genetic stability, cell death, and stem cell renewal (310). The HIF-1α signaling pathway mediates the transcription of genes, allowing cells to adapt to hypoxic environments and lead to changes in glycolysis, nutrient uptake, waste handling, angiogenesis, cell death, and cell migration that may promote tumor survival and metastasis (311). The PI3K/AKT/mTOR pathway plays a vital role in controlling cell survival, metabolism, cell and tumor growth, and protein synthesis in various conditions, including normal physiological processes and pathological states, with a particular emphasis on cancer (312). And p53 signaling acts as a multifunctional transcription factor that activates and represses a growing number of target genes implicated in cell cycle control, apoptosis, programmed necrosis, autophagy, metabolism, stem cell homeostasis, angiogenesis, and senescence (313). Aberrant activation or suppression of these pathways can promote tumorigenesis, angiogenesis, and metastasis. Additionally, EMT is a crucial process in cancer progression, where epithelial cells acquire a mesenchymal phenotype, allowing increased invasion, migration, and metastasis. Furthermore, multiple studies consistently demonstrate the involvement of MMPs (notably MMP1, MMP2, MMP7, and MMP9) in the processes of migration associated with ECM degradation and EMT. The several mechanisms described involve the regulation of EMT-related genes and pathways, contributing to tumor invasiveness and metastatic potential. It’s important to note that these mechanisms are not exclusive to the mentioned disorders but are commonly observed in various types of cancers.

Most of the patients with an inherited enzymatic disorder will receive a supportive, multidisciplinary treatment to alleviate their symptoms and their multisystemic conditions (314–318). However, specific treatments are available for some of these enzymatic disorders. Specifically, in UCDs and some GSDs, liver transplantation is the most effective treatment option (314, 319). Furthermore, multiple clinical trials are investigating treatment options for metabolic disorders such as the administration of recombinant protein (NCT no. 03378531), gene replacement (NCT no. 02991144), and mRNA administration (NCT no. 03767270) (314). For LSDs, a common therapy is substrate reduction, used to inhibit the synthesis of the accumulating macromolecule, or administration of chaperones, which help proteins to fold into their correct conformation. In addition, another common and effective treatment for some LSDs is enzyme replacement therapy (ERT), in which the deficient enzyme is administered intravenously to patients. The recombinant enzyme is taken up by the cells and the accumulated macromolecules are catabolized in lysosomes. ERT works specifically well for LSDs through the mannose-6-phosphate (M6P) receptor, which can bind and transport M6P-enzymes to lysosomes, therefore the intravenously M6P-tagged enzymes can be taken up by cells through the receptor and then delivered to lysosomes where they will catalyze the accumulated substrate (317). Moreover, several of the metabolic enzymes mentioned here seem to influence the efficiency of some chemotherapeutic drugs. The upregulation or downregulation of some genes in various tumors was associated with chemoresistance against some drugs, and depletion or inhibition of the enzymes can contribute to a higher sensitivity to chemotherapeutic drugs.

Nevertheless, there remain significant information gaps in the understanding of the genetics that underlie enzymatic dysfunction in metabolic diseases and cancer. While there is now a rich literature and well-established understanding of the metabolomics of metabolic diseases and of cancer, as well as gene alterations, including mutations, amplifications and loss of heterozygosity, as well as transcriptional alterations, there is only a poor understanding regarding translational regulatory alterations. Transcriptional changes often are not reflected in the proteome due to post-transcriptional regulatory events, including the selective translational regulation of many mRNAs, and targeted protein degradation. Ultimately, there will need to be a concerted effort to begin integrating these many layers of gene control to build a more complete understanding of enzymatic dysfunction in metabolic diseases and cancer. The identification of metabolic enzymes as potential therapeutic targets and biomarkers holds promise for improving cancer therapy and developing new treatment options. Continued research into the interplay between cancer and metabolic enzyme dysregulation will contribute to our understanding of cancer biology and potentially lead to the development of novel therapeutic strategies.

## Author contributions

Conceptualization: TR-F. Design: TR-F and MM. Resources: TR-F, MM, and AK. Writing—original draft preparation: TR-F, MM, and AK. Writing—reviewing and editing: TR-F, MM, and RS. Figures: TR and MM. Supervision: RS. Funding acquisition: TR-F, AK, and RS. All authors have read and approved the submitted version of the manuscript. All authors contributed to the article.
